# A substitution in the pre-S1 promoter region is associated with the viral regulation of hepatitis B virus

**DOI:** 10.1186/s12985-019-1169-x

**Published:** 2019-05-02

**Authors:** Suguru Ogura, Masahiko Tameda, Kazushi Sugimoto, Makoto Ikejiri, Masanobu Usui, Masaaki Ito, Yoshiyuki Takei

**Affiliations:** 1 Department of Gastroenterology and Hepatology, Mie University Graduate School of Medicine, 2-174 Edobashi, Tsu, Mie 514-8507 Japan; 2 Department of Central Laboratory, Mie University Hospital, Tsu, Japan; 3 Department of Hepatobiliary Pancreatic and Transplant Surgery, Mie University Graduate School of Medicine, Tsu, Japan; 4 Department of Cardiology and Nephrology, Mie University Graduate School of Medicine, Tsu, Japan

**Keywords:** Hepatitis B virus, Mutation, Pre-S1 promoter, L protein

## Abstract

**Background:**

Much evidence has demonstrated the influence of Hepatitis B virus (HBV) mutations on the clinical course of HBV infection. As large (L) protein plays a crucial role for viral entry, we hypothesized that mutations in the pre-S1 promoter region might affect the expression of L protein and subsequently change the biological characters of virus.

**Methods:**

Patients infected with genotype C HBV were enrolled for analysis. HBV DNA sequences were inserted into a TA cloning vector and analyzed. To evaluate the effects of mutations in the pre-S1 promoter region, promoter activity and the expression of mRNA and L protein were analyzed using HepG2 cells.

**Results:**

In total, 35 patients were enrolled and 13 patients (37.1%) had a single base substitution in the pre-S1 promoter region; the most frequent substitution was a G-to-A substitution at the 2765th base (G2765A) in the Sp1 region. The HBV viral load showed a negative correlation with the substitution ratio of the Sp1 region or G2765A (r = − 0.493 and − 0.473, respectively). Among those with a viral load ≤5.0 log IU/ml, patients with the G2765A substitution showed a significantly lower HBV viral load than those with the wild-type sequence. HepG2 cells transfected with the G2765A substitution vector showed reduced luciferase activity of the pre-S1 promoter, as well as reduced expression of pre-S1 mRNA and L protein. Furthermore, the G2765A substitution greatly reduced the L protein expression level of vector-produced virus particles.

**Conclusion:**

G2765A substitution in the pre-S1 promoter reduced the expression of L protein and resulted in a low viral load and less severe disease in chronic HBV infections.

## Background

Despite the promotion of vaccination, HBV infection remains one of the leading causes of severe liver disease, such as liver cirrhosis and HCC. According to the World Health Organization, an estimated 257 million people worldwide are currently infected chronically with HBV, and a higher prevalence is found among populations in African and western pacific regions [1]. As of 2011, 1.2 million people in Japan are estimated to be infected with HBV [2], and genotype C has been reported to be the most dominant strain in Japan, accounting for more than 80% of the chronic infections [3].

HBV is a hepatotropic DNA virus that belongs to the *Hepadnaviridae* family. It contains a partially double-stranded relaxed circular DNA of approximately 3200 nucleotides from which four mRNAs are transcribed, i.e., 0.7-, 2.1-, 2.4-, and 3.5-kb mRNAs. The envelope proteins of HBV consist of, in descending order of size, L protein, M protein, and S protein. L protein is translated from the 2.4-kb mRNA, and the M and S proteins are translated from the 2.1-kb mRNA. A polymerase, i.e., a reverse transcriptase lacking proofreading activity, is translated from the 3.5-kb mRNA. The error-prone feature of this polymerase causes a high mutation rate that is estimated to be 10^− 3^ to 10^− 6^ substitutions per replication cycle, which is more than 10-fold higher that of other DNA viruses [4–6]. HBV mutations may be selected for by immune responses or antiviral therapies, and the species with advantages in replication, assembly, secretion, or infectivity become dominant.

Much evidence has demonstrated the influence of HBV mutations on the clinical course of HBV infection. One of the most common mutations of HBV is G1896A at the precore region that converts TGG to TAG, a stop codon, which abolishes the expression of hepatitis B e antigen (HBeAg). Double mutations, A1762T and G1764A, in the basic core promoter region result in decreased HBeAg expression and enhanced viral genome replication; these mutations are frequently found in HBeAg-negative chronic hepatitis patients [7]. These mutations were documented as predictors of HCC risk [8]. Nucleos(t)ide analogues (NAs) are currently used for the treatment of HBV as they can inhibit the reverse transcription of HBV. However, long-term usage of NAs may lead to the development of drug resistance due to selective pressure [9]. In the surface protein region, mutations in the “α” determinant, which is the major target of the neutralizing B cell response, are known to be associated with immune escape [10]. In addition, PreS1 and/or preS2 deletions and preS2 start codon mutations reduce hepatitis B surface antigen (HBsAg) titer [11].

Recently, sodium taurocholate co-transporting polypeptide (NTCP) has been identified as a host receptor of HBV. NTCP is a bile salt transporter that is expressed on the surface of hepatocytes; it binds to the N-terminus of the L protein, which triggers viral entry [12]. In addition to this, L protein has several other biological functions. It is necessary for virion maturation by binding to core particles [13]. Also, the pre-S1 domain contains many T- and B-cell epitopes that play essential roles in the interaction with the immune system [14]. Furthermore, an inappropriate L/S ratio leads to the inhibition of virion secretion [15], and L protein is also involved in the regulation of covalently closed circular DNA (cccDNA) [16]. As described above, L protein is translated from the 2.4-kb mRNA, different from the 2.1-kb mRNA coding the S and M proteins, and its transcription is regulated by the HNF1, HNF3, Sp1, and TATA transcription factors via binding to recognition sequence that are located upstream of the pre-S1 domain [17–19]. These promoters contain specific nucleotide sequences that transcription factors can bind to and increase transcription activity.

Due to the various functions of L protein, we hypothesized that mutations in the pre-S1 promoter region might affect the expression of L protein and subsequently change the biological characters of virus and prognosis of HBV infection. To examine this hypothesis, in this study, we investigated the effects of mutations in the pre-S1 promoter region of HBV on biological characters of virus and prognosis using clinical samples and a cultured cell line.

## Methods

### Patients

Subjects with chronic HBV infection whose HBV DNA was measured at Mie University Hospital between June 2013 and June 2014 were enrolled in this study. Regular blood chemistry tests were performed at the central laboratory of Mie University Hospital. HBV viral loads were also measured at this laboratory by quantitative real-time PCR (qPCR; COBAS TaqMan HBV test v2.0, Roche, Basel, Switzerland). The HBV genotypes were determined by a clinical laboratory testing company (SRL, Tokyo, Japan) and/or by nucleotide sequencing of the pre-S2 region [20]. Aliquots of patient sera were kept at − 30 °C until the HBV DNA sequence analyses.

Only the patients infected with genotype C HBV were enrolled in the study as it is the most dominant genotype in Japan. The other exclusion criteria included: current or past usage of anti-HBV agents, including interferon and NAs; co-infection with hepatitis C virus or human immunodeficiency virus; association with any other liver disease (autoimmune liver disease, etc.); history of chemotherapy or radiation therapy; and current or past usage of any drugs that could affect the patient’s immune status (steroids, immunosuppressants, etc.). We also excluded patients who were diagnosed as having genotype C HBV by the antigenicity of pre-S2 region in the HBsAg (performed by SRL) if the sequence analyses showed conflicting results. In addition, patients with a low HBV viral load (< 1.3 log IU/ml) were also excluded. This study was approved by the Institutional Review Board of Mie University Hospital. Written informed consent was obtained from each patient included in the study. The study protocol conformed to the ethical guidelines of the 1975 Declaration of Helsinki, as reflected in the a priori approval by the institution’s human research committee.

### Sequencing of HBV

HBV DNA was extracted from the serum of each patient using NucleoSpin Virus (Takara Bio, Shiga, Japan) according to the manufacturer’s instructions. The PCR products covering the region 211 bp upstream from the start codon of the pre-S1 region to 95 bp downstream from the start codon of the S region by the primers S1 and S2 (Table 1) were inserted into a pTAC-1 DynaExpress TA PCR cloning vector (BioDynamics Laboratory, Tokyo, Japan). Competent cells (DH-5α, Toyobo, Osaka, Japan) were transformed with the TA cloning vector and spread on agar. After overnight incubation at 37 °C, at least 15 clones were picked up and cultured in Luria-Bertani medium. The plasmids were isolated using a QIAprep Spin Miniprep kit (Qiagen, Venlo, Netherlands). The HBV DNA sequences inserted into the TA vectors were analyzed using an ABI3100 DNA sequencer (Applied Biosystems, Waltham, MA). The consensus sequence was defined by searching the GenBank database using the terms: HBV, genotype C, complete genome, Japan.

**Table 1:** Primers used for PCR

Name	Sequence (5′ to 3′)	Annotation
S1	ATGCCTGCTAGGTTCTATCC	HBV 2637–2656 (sense)
S2	AGACTCTGTGGTATTGTGAGG	HBV 229–249 (antisense)
W1	TTCACCTCTGCCTAATCATCTCATG	HBV 1824–1848 (sense)
W2	AAGTTGCATGGTGCTGGTGAAC	HBV 1801–1822 (antisense)
F1	CGACTCAGGAAACTGCCTGTAAATAGAC	HBV 950–970, pTac1 404–410 (sense)
F2	AGAGCTTGGTGGAATGTTGTG	HBV 4–24 (antisense)
F3	ATTCCACCAAGCTCTGCTAG	HBV 10–29 (sense)
F4	CCAAGCTAGAAGGAAAGAAGTCAGAAGG	HBV 1958–1978, pTac1 498–504 (antisense)
F5	CAGTTTCCTGAGTCGTATTACGCGCTGG	pTac1 391–410, HBV 950–957 (antisense)
F6	TTCCTTCTAGCTTGGCGTAATCATGGTC	pTac1 498–517, HBV 1971–1978 (sense)
L1	AGActcgagCTTGGACAAAGGCATTAAAC	XhoI, HBV 2678–2697 (sense)
L2	AGGagatctGAGGCGCTGCGTGTAGTTTC	BglII, HBV 2787–2806 (antisense)
S3	TGAGTCGTATTACGCGCTGG	pTac1 391–410 (antisense)
m5	AA**A**GCTGGCATTCTATATAAAAGAG	HBV 2763–2787, Sp1 G2765A (sense)
m9	**GCGACATAAG**TTCTATATAAAAGAG	HBV 2763–2787, Sp1 whole nucleotide mutation (sense)
mR	CCACAGAGTATGTAAATAATGCC	HBV 2740–2762 (antisense)

Nucleotides that were substituted with wild type are indicated in bold. Abbreviations: *PCR* polymerase chain reaction, *HBV* hepatitis B virus

### Next-generation sequencing (NGS)

The samples were prepared according to the Ion Amplicon Library Preparation (Fusion Method) protocol of Thermo Fisher Scientific (Waltham, MA). Two pairs of primers were designed. One primer pair had the A adapter sequence (5′- CCATCTCATCCCTGCGTGTCTCCGACTCAG -3′) with a barcode sequence (13 bp for each sample) followed by the proximal end of the target sequence, and the other pair had the trP1 adapter region (5′- CCTCTCTATGGGCAGTCGGTGAT -3′) followed by the distal end of the target sequence. The other fusion primer pair had the adapter sequences A and trP1 swapped. The sense primer sequence for the target sequence was 5′- ATGCCTGCTAGGTTCTATCC -3′, and the anti-sense primer sequence was 5′- ATCTGGATTGTTTGAGTTGG -3′. Ten samples with high HBV viral load (8.2–7.2 log IU/ml) were selected and amplified using each primer pair. The PCR products were purified by Agencourt AMPure XP (Beckman Coulter, Brea, CA) and quantitated using the Agilent DNA 1000 kit (Agilent Technologies, Santa Clara, CA). The PCR products were prepared into an equimolar pool of amplicon libraries at the concentration of 100 pM. The amplicon pool was sequenced on an Ion Personal Genome Machine with the Ion 318 Chip (Thermo Fisher Scientific). FASTQ files were output using Ion Torrent Suite Software (Thermo Fisher Scientific). The sequences were trimmed and filtered using Trimmomatic [21], and aligned to the reference sequence using BWA-MEM [22]. The mutations in the Sp1 region were detected using Integrative Genomics Viewer [23].

### Vector constructs

The whole HBV DNA genomes isolated from patient sera were amplified by PCR using primers W1 and W2 (Table 1). The amplicons were inserted into the pCR4 Blunt TOPO vector (Thermo Fisher Scientific) and sequenced. One clone that had no mutation in the pre-S1 promoter region was selected as a template. A plasmid containing the SapI restriction enzyme recognition sequence was constructed according to a previously reported method [24]. After the plasmid was digested by SapI, it was self-ligated with T4 DNA ligase to make a full-length circular HBV structure. Using this structure as a template, a plasmid containing a 1.3-fold HBV genome was constructed according to a previously described method [25] with modifications as follows. Circular HBV DNA was amplified by PCR using the primer pairs F1 and F2, and F3 and F4, and the pTac1 TA cloning vector was amplified using primers F5 and F6 (Table 1). The three amplicons were ligated using the In-Fusion HD Cloning Kit (Takara Bio) to create a 1.3-fold HBV genome vector construct, which was transformed into competent cells (pTac-1 1.3-fold HBV genome).

Luciferase vectors were constructed using the following methods. PCR products that covered the region 170 bp upstream from the start codon of the pre-S1 region to 41 bp upstream from the start codon of the pre-S1 region were amplified with primers L1 and L2 (Table 1), which also added a sequence recognized by the XhoI or BglII restriction enzyme, respectively. The amplicon and pNL1.1 [Nluc] vector (Promega, Madison, WI) were digested by XhoI and BglII, then ligated and transformed into competent cells (pNL1.1[Nluc] pre-S1 promoter). The L protein expression vector was constructed by an inverse PCR method [26]. The 1.3-fold HBV genome vector was amplified with primers S1 and S3 (Table 1). The amplicon covered the region 211 bp upstream from the start codon of the pre-S1 region to the poly-A signal region was circularized using T4 ligase, then transformed into competent cells (pTac-1 pre-S1 expression).

**Fig. 1:**
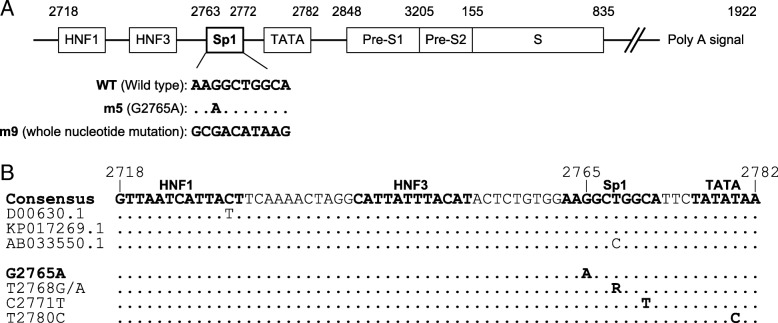
Schematic representation showing the structure of the vector constructs and pre-S1 promoter sequence. **a** A schematic diagram showing the structure of the pre-S1 promoter and the wild type (WT), G2765A (m5), and whole nucleotide mutation (m9) nucleotide sequences of the Sp1 region. **b** Alignment of the pre-S1 promoter nucleotide sequences, including the consensus sequence, representative references, and substitution strains. Nucleotide symbol R indicates A (adenine) or G (guanine)

The mutation vectors were constructed by the inverse PCR method using primer m5 (G2765A), a sense primer for the G-to-A substitution at the third nucleotide of the Sp1 region, and primer m9 (whole nucleotide mutation), a sense primer for a whole nucleotide mutation of the Sp1 region, and antisense primer mR (Table 1). The pNL1.1[Nluc] pre-S1 promoter, pTac-1 pre-S1 expression, and pTac-1 1.3-fold HBV genome vectors were amplified using these primers pairs, and the amplicons were circularized using T4 ligase, then transformed into competent cells. The structures of the vectors are detailed in Fig. 1a.

### Cell culture and vector transfection

The human HCC cell line HepG2 (RCB1648) was purchased from the RIKEN BRC Cell Bank (Tsukuba, Japan). The cells were cultured in Dulbecco’s modified Eagle’s medium (Life Technologies, Carlsbad, CA) supplemented with 1% penicillin/streptomycin (Life Technologies) and 10% fetal calf serum (Life Technologies) in a humidified atmosphere containing 5% CO2 at 37 °C.

HepG2 cells were seeded in a 6-well plate (1 × 10^6^ cells/well) and incubated for 24 h before transfection. The cells were transfected with 2.5 μg of pre-S1 expression vector or the 1.3-fold HBV genome vector using Lipofectamine 3000 reagent (Thermo Fisher Scientific). Protein samples and mRNA samples were collected 48 h and 24 h after transfection, respectively.

### Luciferase assay

HepG2 cells were seeded in a 96-well plate (3 × 10^4^ cells/well) and incubated for 24 h before transfection. The cells were transfected with 50 ng of Sp1 promoter-inserted pNL1.1 vectors and 50 ng of a pGL4.54 TK promoter control firefly luciferase vector. The cells were analyzed 24 h after transfection using the Nano-Glo Dual-Luciferase Reporter Assay System (Promega) according to the manufacturer’s instructions. The levels of NanoLuc luciferase activity were determined by normalization to the levels of firefly luciferase activity.

### RNA extraction and qPCR

Total RNA was extracted from cultured cells with the RNeasy Mini Kit and RNase-Free DNase Set (Qiagen, Hilden, Germany), and polyA^+^ mRNA was purified using Oligotex™ -dT30<Super> mRNA Purification Kit (Takara Bio). cDNA was synthesized from 10 ng of mRNA by the extension of oligo dT and random 6 mer primers with the PrimeScript RT reagent kit (Takara Bio). Subsequently, qPCR was performed using the ABI Prism 7300 Real-Time PCR System (Applied Biosystems, Foster City, CA) with EagleTaq Master Mix kits (Roche Molecular Systems, Branchburg, NJ). The primer and probe sets used for the amplification of the pre-S1 2.4-kb mRNA were 5′- aggctcagggcatattgaca − 3′, 5′- gtcttcctgactgccgattg − 3′, and Universal ProbeLibrary Probe #16 (Roche). The expression levels of the target gene were determined by normalization to β-actin by the following primers: 5′- aagtcccttgccatcctaaaa − 3′, 5′- atgctatcacctcccctgtg − 3′.

### Western blot analysis

Proteins of the cell lysates and cell culture supernatants were separated by sodium dodecyl sulfate polyacrylamide gel electrophoresis and transferred onto polyvinylidene difluoride membranes. The blots were blocked with 5% milk in Tris-buffered saline with 0.1% Tween-20 for 1 h, then probed with rabbit polyclonal anti-β-actin antibody (diluted 1:4000; Abcam, Cambridge, UK), mouse monoclonal anti-HBs antibody (diluted 1:2000; Cosmo Bio, Tokyo, Japan) or mouse monoclonal anti-HBV pre-S1 antibody (diluted 1:4000; Beacle, Kyoto, Japan) at 4 °C overnight. The immunoblots were then probed with horseradish peroxidase-conjugated anti-mouse secondary antibody (diluted 1:4000; GE Healthcare, Amersham, UK) and visualized using Amersham ECL Prime Western Blotting Detection Reagent (GE Healthcare). Protein amounts were determined by densitometry.

### Statistical analysis

Comparisons of the luciferase activity, mRNA expression, and protein production were performed using the unpaired t-test for comparisons between two groups, one-way ANOVA for comparisons between three groups, or Fisher’s exact test for comparisons of categorical variables. Comparison of the clinical parameters was performed using the Mann-Whitney test. The interdependence between two parameters was examined using Spearman’s correlation coefficient. *P* < 0.05 was considered to be statistically significant.

## Results

### Definition of the consensus sequence and vector construction

Based on the search of the GenBank database, the consensus sequence of the pre-S1 promoter region was defined as shown in Fig. 1b. The full genomic sequence of the HBV DNA strain obtained from a patient that had no mutation in the pre-S1 promoter region and that mostly matched with the consensus sequence was determined and deposited into the DNA Data Bank of Japan under accession number LC360507. This strain was used as a template to construct the vectors.

### Identification of the mutations in the pre-S1 promoter region

In total, 35 patients were enrolled in this study. To assess the mutation in each pre-S1 promoter region, we picked up at least 15 clones from each patient and compared them to the consensus sequence using DNASIS Pro Ver. 2.9 software (Hitachi, Tokyo, Japan); we defined cases in which more than 50% of the clones had a mutation as cases with a mutation. According to these analyses, 13 patients (37.1%) had single base substitutions that were detected in the Sp1 and TATA regions of the pre-S1 promoter; the characteristics of these patients are shown in Table 2. Substitutions in the Sp1 region were found at the 2765th base (G to A; G2765A), 2768th base (T to G or A), and 2771st base (C to T). A substitution in the TATA region was found at the 2780th base (T to C; Fig. 1a). These four substitutions were silent mutations for the polymerase of HBV.

**Table 2:** Characteristics of patients with pre-S1 promoter mutant or wild-type HBV

	All (*n* = 35)	Wild type (*n* = 22)	Mutation (*n* = 13)	p
Male (n (%))	20 (57.1%)	10 (45.5%)	10 (76.9%)	0.089
Age (years)	51.7 (15–81)	49.2 (15–81)	55.9 (29–74)	0.432
HBV DNA (log IU/ml)	5.11 (1.3–8.2)	5.22 (1.3–8.2)	2.86 (1.3–8.2)	0.003
HBsAg (IU/ml)	13,499 (8.61–151,233)	17,788 (8.61–151,233)	5634 (37.7–19,986)	0.094
HBeAg positive (n (%))	11 (31.4%)	10 (45.5%)	1 (7.7%)	0.027
HBeAb positive (n (%))	24 (68.6%)	12 (54.5%)	12 (92.3%)	0.027
AST (U/L)	34.5 (11–204)	41.4 (16–204)	22.7 (11–40)	0.035
ALT (U/L)	43.8 (10–425)	56.5 (14–425)	22.4 (10–49)	0.127
Platelet count (10^4^/μl)	18.5 (10.9–24.2)	18.8 (13.9–23.9)	18.0 (10.9–24.2)	0.705

Abbreviations: *HBV* hepatitis B virus, *HBsAg* hepatitis B surface antigen, *HBeAg* hepatitis B e antigen, *HBeAb* hepatitis B e antibody, *AST* aspartate transaminase, *ALT* alanine transaminase

### Comparison of viral sequence data acquired by cloning and NGS

To confirm the integrity of the viral sequence data obtained from cloning, we picked up 10 samples and performed NGS analyses to compare the data derived from both methods. As shown in Table 3, the ratios of the Sp1 G2765A substitution for each sample were mostly comparable between the cloning and NGS data, which verified that our cloning sequence data were reliable.

**Table 3:** G2765A substitution ratios determined by TA cloning or next-generation sequencing

TA cloning^a^	Substitution percentage	NGS^a^	Substitution percentage
1/15	6.67%	44/6155	0.71%
0/15	0.00%	1/3955	0.03%
0/15	0.00%	10/5327	0.19%
9/15	60.00%	4866/8081	60.22%
0/16	0.00%	7/10409	0.07%
0/15	0.00%	2/7304	0.03%
0/15	0.00%	9/6662	0.14%
1/25	4.00%	22/6258	0.35%
1/16	6.25%	49/1643	2.98%
0/15	0.00%	1/2740	0.04%

These samples were selected from high HBV viral load (8.2–7.2 log IU/ml) patients

^a^ Number of samples with the G2765A substitution/total number of samples

Abbreviation: *NGS* next-generation sequencing

### Relationships between mutations in the pre-S1 promoter region and clinical parameters

**Fig. 2:**
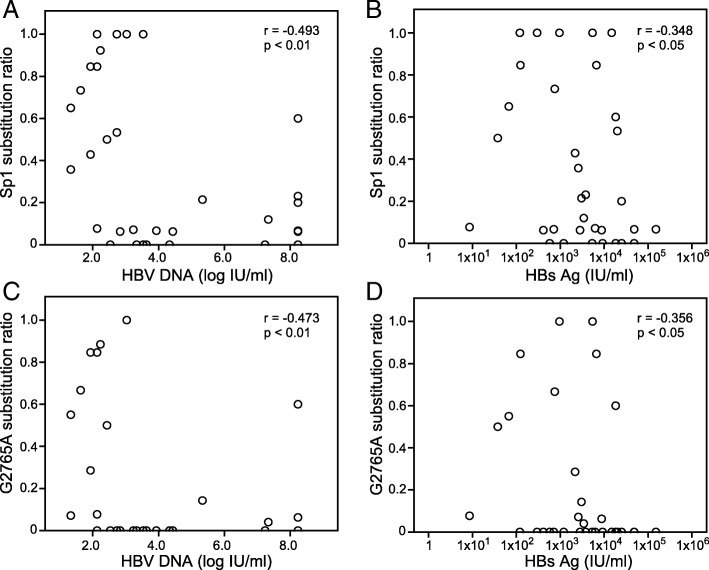
Correlation between the substitution ratio and HBV-related parameters. **a** The Sp1 substitution ratio and the HBV DNA viral load showed a negative correlation, and (**b**) the Sp1 substitution ratio and the HBsAg level showed a weak negative correlation. **c** The G2765A substitution ratio and the HBV DNA viral load showed a negative correlation, and (**d**) the G2765A substitution ratio and the HBsAg level showed a weak negative correlation

We analyzed whether the mutations in the pre-S1 promoter region affected the clinical parameters. When compared to the patients who had no mutation, the HBV viral load was significantly lower (*p* < 0.01) in those with a mutation; the level of HBsAg was also lower in those with a mutation, but not to the point of statistical significance (Table 2). The Sp1 substitution ratio and the HBV viral load showed a negative correlation (*r* = − 0.493, *p* < 0.01), and the Sp1 substitution ratio and the HBsAg level showed a weak negative correlation (*r* = − 0.348, *p* < 0.05; Fig. 2a and b). It is conceivable that a Sp1 substitution is associated with the HBV viral load, so we divided patients into two groups according to the HBV viral load: a high group whose viral load was > 5.0 log IU/ml and a low group whose viral load was ≤5.0 log IU/ml; 11 patients were classified into the high group and 24 patients were classified into the low group. Twelve patients in the low group and one patient in the high group had a Sp1 substitution, and the Sp1 substitution ratio was significantly higher in the low group than in the high group (Table 4, *p* < 0.05). The substitution in the TATA region was detected in one patient in the low group. The most frequent substitution was the G2765A substitution, which was found in one patient in the high group and eight patients in the low group. In the low group, the HBV viral load was lower in the patients who had a Sp1 substitution than in those who had no mutation, but the difference was not statistically significant (Table 5). On the other hand, patients who had the G2765A substitution showed a significantly lower HBV viral load and aspartate transaminase (AST) level when compared to those with the wild-type (WT) sequence (*p* < 0.05). In addition, the G2765A substitution ratio and the HBV viral load showed a negative correlation (*r* = − 0.473, *p* < 0.01), and the G2765A substitution ratio and the HBsAg level showed a weak negative correlation (*r* = − 0.356, *p* < 0.05; Fig. 2c and d). Therefore, we focused on the G2765A substitution and analyzed its biological function.

**Table 4:** Occurrence of substitutions at different sites among patients with a high (> 5.0 log IU/ml) or low (≤5.0 log IU/ml) HBV DNA viral load

	High (*n* = 11)	Low (*n* = 24)	p
HNF1	0	0	> 0.999
HNF3	0	0	> 0.999
Sp1	1	12	0.027
G2765A	1	8	0.219
T2768G/A	0	2	> 0.999
C2771T	0	2	> 0.999
TATA	0	1	> 0.999
T2780C	0	1	> 0.999

**Table 5:** Characteristics of patients in the low HBV viral load group according to Sp1 and the G2765A substitution

		Sp1		G2765A	
	Wild type (*n* = 12)	Substitution (*n* = 12)	p	Substitution (*n* = 8)	p
Male (n (%))	4 (33.3%)	9 (75.0%)	0.1	6 (75.0%)	0.170
Age (years)	53.9 (35–81)	58.2 (32–74)	0.522	63.4 (46–74)	0.150
HBV DNA (log IU/ml)	3.9 (1.3–4.4)	3.2 (1.3–3.5)	0.061	2.2 (1.3–3.0)	0.040
HBsAg (IU/ml)	4129 (8.61–18,268)	4483 (37.7–19,986)	0.525	2007 (37.7–6670)	0.340
HBeAg positive (n (%))	0 (0%)	0 (0%)	> 0.999	0 (0%)	> 0.999
HBeAb positive (n (%))	12 (100%)	12 (100%)	> 0.999	8 (100%)	> 0.999
AST (U/L)	26.8 (19–50)	22.0 (11–40)	0.074	19.0 (11–24)	0.011
ALT (U/L)	31.9 (14–90)	20.2 (11–40)	0.235	18.8 (10–27)	0.162
Platelet count (10^4^/μl)	17.7 (13.9–22.5)	17.4 (10.9–22.6)	0.921	17.5 (10.9–22.6)	0.955

Abbreviations: *HBV* hepatitis B virus, *HBsAg* hepatitis B surface antigen, *HBeAg* hepatitis B e antigen, *HBeAb* hepatitis B e antibody, *AST* aspartate transaminase, *ALT* alanine transaminase

### A substitution in the Sp1 site reduced the transcription of pre-S1

**Fig. 3:**
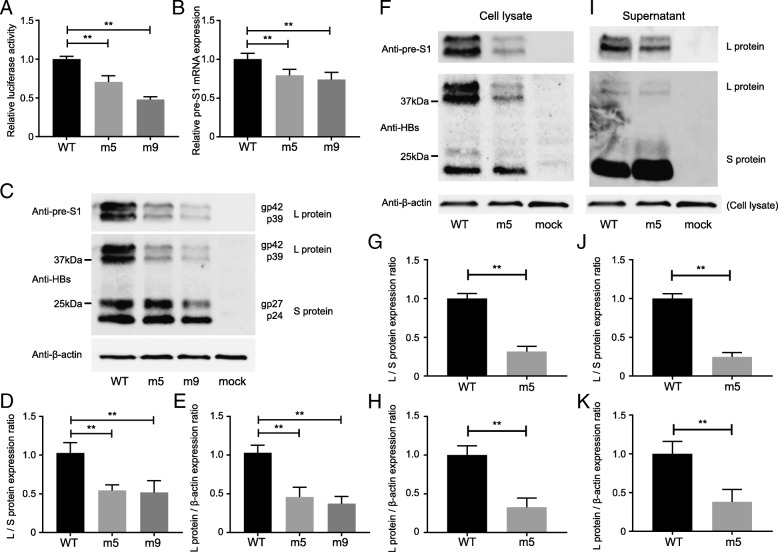
A mutation in the pre-S1 promoter region Sp1 reduced transcription activity: (**a-e**) The levels of luciferase activity, mRNA expression, and protein expression of pre-S1 were evaluated using vectors containing the wild-type (WT), G2765A substitution (m5), and whole nucleotide mutation (m9) sequences. **a** pNL vectors inserted with the Sp1 promoter and a pGL4.54 TK promoter control vector were transfected into HepG2 cells. The levels of luciferase activity were normalized to the levels of the control vector. **b** Pre-S1 expression vectors were transfected into HepG2 cells. After 24 h, the total RNA was extracted, and polyA+ mRNA was purified and measured by qPCR. **c-e** Forty-eight hours after transfection with the pre-S1 expression vector, cell lysates were extracted and evaluated by immunoblotting using anti-HBs, anti-pre-S1 and anti-β-actin antibodies. The levels of L protein expression were normalized to the levels of S protein expression (**d**) or β-actin expression (**e**). **f-k** The wild-type (WT) or G2765A (m5) 1.3-fold HBV genome vector was transfected into HepG2 cells. The (**f-h**) cell lysates and (**i-k**) supernatants were evaluated by immunoblotting using anti-HBs, anti-pre-S1 and anti-β-actin antibodies. The levels of L protein expression were normalized to the levels of S protein expression (**g, j**) or β-actin expression (**h, k**). β-actin level of cell lysate was used for normalization of the supernatant sample isolated from the same well

We hypothesized that a substitution in the Sp1 site upstream of the pre-S1 region could affect the transcription of the mRNA coding large HBsAg. To confirm this hypothesis, we first performed a luciferase assay using HepG2 cells. Both m5 (G2765A) and m9 (whole nucleotide mutation) vectors showed significantly impaired luciferase activity (70.7 and 47.9%, respectively) when compared to the WT vector (*p* < 0.01; Fig. 3a), suggesting that a substitution in Sp1 caused the reduced transcriptional activity of pre-S1 mRNA.

To further investigate whether a substitution in the Sp1 site could influence the expression of L protein, we analyzed the changes in pre-S1 mRNA and L protein expression using pre-S1 expression vectors. First, we analyzed pre-S1 mRNA and L protein expression by using an expression vector that included the transcription-regulating regions to the end of the S region. Pre-S1 mRNA expression was significantly decreased in the cells transfected with the m5 or m9 vector when compared to those transfected with the WT vector (79.4%, *p* < 0.01 and 74.1%, *p* < 0.01, respectively; Fig. 3b). The expression of L protein was significantly decreased in the cells transfected with the m5 or m9 vector when compared to those transfected with the WT vector (*p* < 0.01, respectively; Fig. 3c-e). These results clearly indicated that a substitution in the Sp1 site reduced the transcription of the L protein.

### The Sp1 G2765A substitution reduced the level of pre-S1 antigen

To further investigate the effect of the G2765A substitution on HBV production, we constructed a 1.3-fold HBV genome vector that enabled the production of HBV particles when transfected into HepG2 cells. Transfection of the m5 (G2765A) or WT vector into HepG2 cells resulted in no change in virus production (5.6 log IU/ml, 5.7 log IU/ml, respectively). However, the expression of the L protein was significantly decreased in both the cell lysate (Fig. 3f-h) and supernatant (Fig. 3i-k) of HepG2 cells transfected with the m5 vector when compared to those transfected with the WT vector (*p* < 0.01, respectively).

## Discussion

In the present study, we found that there were several mutation sites in the pre-S1 promoter region of HBV, among which Sp1 was the most common mutation site. In addition, patients who had a mutation in the pre-S1 promoter region showed less severe disease (for example, lower aminotransferase levels, higher rate of HBeAb positivity, and lower HBV viral load) than those who did not, and the Sp1 substitution ratio was negatively correlated with the HBV amount. Moreover, our in vitro study using cultured cells showed that the expression of L protein was significantly decreased by a single nucleotide change from G to A at the 2765th base in the Sp1 site.

It has been reported that the risk of chronic hepatitis B progression is associated with several HBV viral factors, including the viral load, HBsAg level, and HBV genotype. Patients with a high viral load or high HBsAg level are reported to have a higher risk of HCC, especially in patients with genotype C when compared to those with genotype B [8, 27]. In contrast, patients with a low HBV DNA load and low HBsAg level have a lower risk of disease progression, and these are associated with HBsAg seroclearance [28, 29]. Furthermore, HBV mutations affect the prognosis of HBV patients. The A1762T and G1764A mutations in the basic core promoter region are associated with an increased risk of HCC in genotype C patients [8]. Based on the above, we hypothesized that a mutation in the pre-S1 promoter region might affect the prognosis of HBV infection.

Our data from the clinical samples showed that the G2765A substitution was associated with a lower HBV DNA load. We think the most probable explanation for this is that the substitution reduces the production of L protein, which plays a crucial role in the virion secretion [15]. Our transfection experiments using cultured cells showed that a mutation in the Sp1 site significantly reduced the transcription of L protein mRNA and L protein production, lowering the L/S protein ratio. These results suggest the possibility that a mutation in Sp1 promoter region reduces the expression of L protein, consequently inhibiting virion secretion, thus leading to a lower viral load and silent disease in the long run.

Nevertheless, it remains unclear how HBV with a mutation in the pre-S1 promoter region could become dominant even though it is likely less infectious to hepatocytes due it the lower viral load. We speculated that the reason might be related to the immune response. The envelope proteins of HBV are immunogenic, and L protein contains both B- and T-cell epitopes and plays an important role in the immune responses [14]. HBV is constantly exposed to host immune pressure, and HBV with mutations that allow it to escape from that pressure are able to survive. Reduced levels of L protein caused by the G2765A substitution may enable HBV to escape immune pressure, making it possible to persist in the hepatocytes of patients. In fact, the aminotransferase levels of patients infected with the G2765A mutant HBV tend to be low, indicating that the immune responses of those patients are weak.

Our findings may also shed light on how a decrease in L protein level allows HBV to remain alive in hepatocytes. Much evidence has been reported on the role of L protein in several stages of HBV production. Garcia et al. proposed that either a sufficiently high or low L/S protein ratio inhibited virion secretion [15], and this was corroborated by our finding that patients infected with the G2765A mutant HBV showed a lower HBV DNA load. In addition, Lentz et al. argued that ablation of the expression of envelope proteins causes an increase in cccDNA, and L protein regulates the amplification of cccDNA [16]. Our results suggest the possibility that the G2765A substitution reduced L protein levels, leading to an increase in the number of cccDNA, which is difficult to eliminate from hepatocytes. It’s as if HBV has “gone underground like the Resistance” for survival. However, the relationship between the cccDNA level and the prognosis of HBV-infected patients remains poorly understood. In fact, G2765A mutant HBV-infected patients showed less severe disease. Therefore, the G2765A substitution is considered to serve as a marker of good prognosis.

The present study has several limitations. First, it was performed with a relatively small number of patients. The reason for this was the very strict inclusion criteria used for the study. We focused only on genotype C-infected patients, because it is the genotype that is mainly found in East Asia, and more than 80% of chronic HBV-infected patients have genotype C HBV in Japan [3]. In addition, genotype C HBV infection is more burdensome than HBV infection with the other genotypes, because it is associated with a higher risk of cirrhosis and HCC, and tends to be resistant to antiviral therapy. In clinical settings, HBV genotypes are usually determined serologically using commercial enzyme immunoassay (EIA) kits that detect the pre-S2 epitope. However, different HBV genotypes can simultaneously infect the same host, and in that situation, the results of EIA may be scrambled; as such, in this study, we confirmed the genotype by both EIA and viral sequencing, and we excluded the subjects for whom the genotyping results were inconsistent. Furthermore, we also excluded the patients who had chronic co-infection with another virus, other liver diseases, or a history of using any kind of medications that could influence the viral and immune status (for example, nucleotide analogues, interferon, immunosuppressants, steroids, anticancer agents, biologic agents, etc.). These strict inclusion/exclusion criteria resulted in a small sample size; however, we believe it also made our data very solid and reliable. Another limitation of the study was our failure to clarify whether HBV with the G2765A substitution is actually less infectious to hepatocytes, and we could not directly show whether the mutation is associated with a decrease in the L/S protein ratio in patient sera due to technical reasons. In addition, we could not perform a long-term survey to prove whether the mutation occurred prior to the reduction in HBV DNA. Further studies are needed for addressing these issues.

## Conclusion

In conclusion, despite several limitations, our present study demonstrated for the first time the possibility that mutations in the pre-S1 promoter region, especially the G2765A substitution in the Sp1 region, reduce the expression of L protein and regulate biological character of virus, leading to a low viral load and less severe disease in chronic HBV infection.

## Abbreviations

AST: Aspartate transaminase
cccDNA: Covalently closed circular DNA
EIA: Enzyme immunoassay
HBeAg: Hepatitis B e antigen
HBsAg: Hepatitis B surface antigen
HBV: Hepatitis B virus
NA: Nucleos(t)ide analogue
NGS: Next-generation sequencing
NTCP: Sodium taurocholate co-transporting polypeptide
qPCR: Quantitative real-time polymerase chain reaction
